# Synchronous Rendezvous for Networks of Marine Robots in Large Scale Ocean Monitoring

**DOI:** 10.3389/frobt.2019.00076

**Published:** 2019-09-04

**Authors:** Xi Yu, M. Ani Hsieh, Cong Wei, Hebert G. Tanner

**Affiliations:** ^1^ScalAR Lab, GRASP Lab, Department of Mechanical Engineering and Applied Mechanics, School of Engineering and Applied Science, University of Pennsylvania, Philadelphia, PA, United States; ^2^Department of Mechanical Engineering, University of Delaware, Newark, DE, United States

**Keywords:** synchronous rendezvous, mobile sensor networks, multi-agent systems, optimal control, consensus

## Abstract

We develop a synchronous rendezvous strategy for a network of minimally actuated mobile sensors or *active drifters* to monitor a set of Lagrangian Coherent Structure (LCS) bounded regions, each exhibiting gyre-like flows. This paper examines the conditions under which a pair of neighboring agents achieves synchronous rendezvous relying solely on the inherent flow dynamics within each LCS bounded region. The objective is to enable drifters in adjacent LCS bounded regions to rendezvous in a periodic fashion to exchange and fuse sensor data. We propose an agent-level control strategy to regulate the drifter speed in each monitoring region as well as to maximize the time the drifters are connected and able to communicate at every rendezvous. The strategy utilizes minimal actuation to ensure synchronization between neighboring pairs of drifters to ensure periodic rendezvous. The intermittent synchronization policy enables a locally connected network of minimally actuated mobile sensors to converge to a common orbit frequency. Robustness analysis against possible disturbance in practice and simulations are provided to illustrate the results.

## 1. Introduction

There is much interest in using networked distributed robotic systems for large-scale environmental monitoring applications, such as coastal surveillance, scientific data collection, and surveying for ocean mining (Yuh et al., 2011; Zhang et al., 2015). Swarms of marine robots can cover large areas and simultaneously collect, process, and interpret data at various distinct geographic locations of interest over prolonged periods of time. Nevertheless, these vehicles must operate with finite power budgets and thus it is extremely important to consider energy aware control and coordination strategies for any data harvesting, exchange, and upload applications.

In this work, we consider the teams of networked minimally actuated drifters or similarly power-constrained mobile sensors that must leverage the dynamics of the ocean flow in order to minimize consumption during navigation. These *active drifters* are limited to intermittent and short-range interactions, which gives rise to a particular type of dynamic and sparse sensor network. This network stays disconnected for most of the time, and has brief periods in which small, isolated units come within communication range and form cliques in the network. Questions of interest here are under which conditions such cliques are formed, how frequently do they appear, how could information propagate if they share some members, and how can the formation of such cliques be made more robust, given that the nodes can only interact with each other when they are in close proximity, i.e., within communication range.

Motion plans and control strategies for robots that are part of a mobile sensor network needs to capture the interplay between sensing, communication, and mobility. Existing work has mostly focused on enabling robots to efficiently harvest and transport data from stationary sensors deployed across large geographical regions (Bhadauria et al., 2011; Sugihara and Gupta, 2011). This is typically done by tasking robots to assist in the data exchange between sensor nodes by physically downloading, carrying, and uploading data from one node to another. Such an approach minimizes the transmission power needed at each node as well as the number of relay nodes in the network. Recent work (Zavlanos, 2010) considered the synchronous arrival of pairs of robots at predefined set of stationary rendezvous points, which was coined as the *synchronous rendezvous* problem. Zavalanos developed distributed agreement protocols such that robots travel between its two adjacent rendezvous points and wait for a finite time upon arriving one of them to rendezvous with its ineighbors.

**Figure 1:**
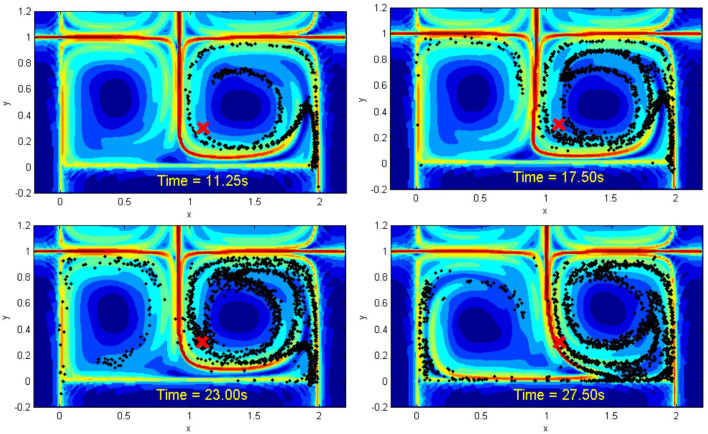
Simulation of a contaminant spill in a time-varying wind-driven double-gyre flow. The LCS boundaries are shown as red curves and the red *x* denotes the source position of the spill. Black particles denote particulates emanating from the source. The center vertical LCS boundary oscillates horizontally about *x* = 1.

**Figure 2:**
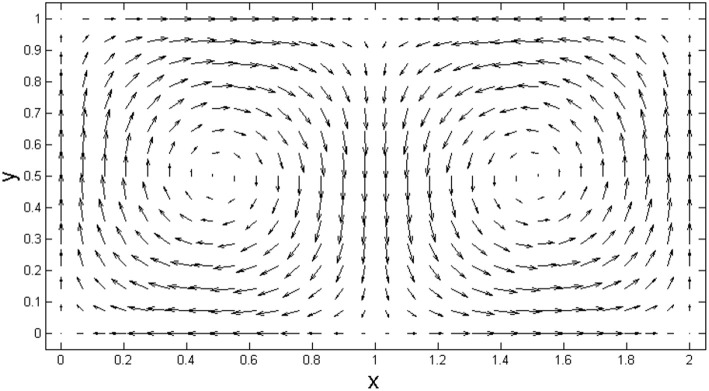
Phase portrait of the wind-driven double-gyre model at *t* = 0.

In this work, we observe that synchronous rendezvous between agents in the ocean-like flows is a variant of the non-linear oscillator synchronization problem. However, since robot motions are dictated by the geophysical fluid dynamics, the synchronized arrival of these mobile sensors must rely on motion plans and control strategies that are *in concert* with the ocean current patterns. As such, the strategy of waiting at a given rendezvous location (Zavlanos, 2010) would be too power intensive to achieve with power-constrained vehicles. Instead, our work leverages the geophysical fluid dynamics in the selection of candidate rendezvous locations and the synthesis of the autonomous vehicle control strategy. We employ a tessellation of the workspace along Lagrangian coherent structures (LCS). LCS are material lines that organize fluid-flow transport and can be viewed as the extensions of stable and unstable manifolds to general time-dependent systems (Haller, 2011). In two-dimensional (2D) flows, LCS are one-dimensional separating boundaries analogous to ridges defined by local maximum instability and can be quantified by local measures of Finite-Time Lyapunov Exponents (FTLEs) (Shadden et al., 2005; Haller, 2011). Recently, LCS have been shown to correspond to minimum energy and time optimal paths in the ocean (Inanc et al., 2005). Despite being global features of the flow field, it has been shown that LCS can be tracked in real time by teams of autonomous vehicles using only local measurements of the flow velocity (Hsieh et al., 2015). Figure 1 shows a simulation of the dispersion of particulates in a time-varying wind-driven double-gyre flow where the LCS boundaries are marked as red curves and the corresponding velocity field is shown in **Figure 2**. Figure 1 suggests that (a) Lagrangian Coherent Structure (LCS) boundaries behave as basin boundaries, and thus fluid from opposing sides of the boundary do not mix; (b) in the presence of noise^1^, particles can cross the LCS boundaries, and thus LCS denote regions in the flow field where more escape events occur (Forgoston et al., 2011); and (c) it makes sense to decompose the oceanic workspace along LCS boundaries and assign sensors to each LCS-bounded region for large-scale monitoring operations (Hsieh et al., 2014).

**Figure 3:**
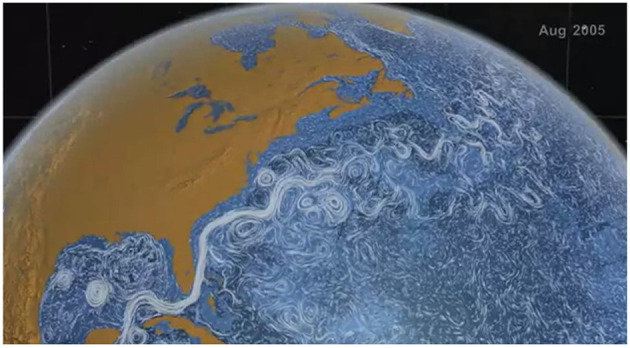
Snapshot (August 2005) of visualization of ocean surface currents for June 2005 through December 2007 generated using NASA/JPLs Estimating the Circulation and Climate of the Ocean, Phase II (ECCO2) ocean model.

While the model shown in Figures 1, 2 presents an idealized representation of the flow field, a snapshot of the ocean surface currents in August 2005 (Figure 3) shows a variety of flow patterns including jets and gyres similar to those in Figures 1, 2,^2^. The states of the loop current can be extracted from sea surface height data (Zeng et al., 2015; Liu et al., 2016). In fact, the time-varying wind-driven double-gyre model is often used to model large-scale ocean circulation (Veronis, 1966).

Leveraging our understanding of LCS, we assume that the workspace can be modeled as a collection of LCS bounded regions exhibiting gyre-like flows. Decomposing the workspace along LCS boundaries allows mobile sensors to leverage the surrounding fluid dynamics for navigation, thus enabling an energy aware control strategy (Kularatne et al., 2018; Wei et al., 2019). Within this geophysical fluid context, the synchronous rendezvous problem can then be mapped to a problem akin to the synchronization of networked oscillators often found in physics, biology, neuroscience, and engineering (Buck and Buck, 1968; Shuai and Durand, 1999; Pikovsky et al., 2001). Nevertheless, existing strategies for distributed oscillator synchronization does not map exactly to the problem of persistent sensing and continuous monitoring by networks of agents subject to spatiotemporal-dependent and intermittent communication (Gazi and Passino, 2003; Sepulchre et al., 2004; Papachristodoulou and Jadbabaie, 2006). Existing strategies do not allow for the *a priori* prediction of the equilibrium consensus state and existing phase or location synchronization. As such, the work presented in this paper addresses the coordination problem through *phase* or *location* synchronization.

This paper builds upon our preliminary work (Wei et al., 2018a) and contributes new synchronous rendezvous strategies and analyses on the conditions for rendezvous for any pair of agents undergoing periodic motion in 2D or 3D flows. In Wei et al. (2018a), we provided strategies for a team of robots to synchronize their frequencies and realize periodic rendezvous. However, noise and disturbance in the system may drift the agents from their synchronized frequencies and result in: (i) some agents may miss their neighbors in a scheduled rendezvous or (ii) some of the agents' rendezvous may be delayed for too long, and as such is not applicable in the real world. In this work, we addressed both issues by analyzing the robustness of the overall synchronization strategy and the susceptibility of network to uncertainties. Our results show that it is *very rare* for pairs of sensors in gyre-like flows never rendezvous and for synchronized pairs of sensors lose their synchronicity in the presence of noise or disturbances. The resulting robust strategy is only possible due to the careful synthesis of ideas from non-linear dynamics, transport theory, and distributed control.

The rest of the paper is organized as follows: section 2 offers a more complete problem statement, while section 3 presents the analysis of the synchronous rendezvous conditions for a single pair of agents, and section 4 provides the synthesis of the short-range coordination strategies. Section 5 analyzed the effect of disturbance in the input. Section 6 presents simulation results. Conclusions and final thoughts close the paper in section 7.

## 2. Problem Formulation

Let the workspace W be composed of *N* adjacent non-overlapping gyres similar to Figures 2, 3. Motions within each gyre can be abstracted to *N* non-overlapping circular orbits in ℝ^2^ indexed by *i* ∈ {1, …, *N*}. Each robot or active drifter deployed within a gyre is assumed to have limited but enough control authority to keep it on its designated circular orbit. Thus, each agent travels along its corresponding orbit continuously and has the same index as its orbit. In addition, each active drifter is only capable of local communication and thus has a finite communication range. The position of agent *i* on its orbit at time *t* is denoted as *x*_*i*_(*t*) and can be represented as the phase of an oscillator θ_*i*_(*t*) ∈ (−π, π]. Let ϕ_*i*_ = θ_*i*_(0) denote the initial phase, then the single vehicle dynamics is given by

(1) $$\begin{array}{l} {\overset{∙}{\theta }}_{i}\left(t\right)=\omega_{i}+u_{i}\left(t\right), \end{array}$$

where ω_*i*_ denotes the natural frequency where agents move along the orbit and *u*_*i*_(*t*) denotes the control input. In general, if *u*_*i*_(*t*) ≡ 0 the agent's natural period would be given by τ_*i*_. The agent can maintain a desired fixed period *T*_*i*_ if there exists a mapping of *u*_*i*_ = *f*(θ_*i*_). Notice that any flow that allows an agent to travel on a closed curve can be modeled as such circular orbits.

**Figure 4:**
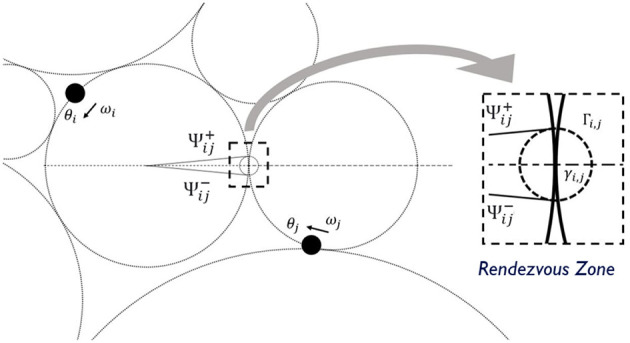
The details of a rendezvous zone Γ_*i,j*_ with the entering and the exiting phases ${\text{Ψ}}_{i,j}^{-}$ and ${\text{Ψ}}_{i,j}^{+}$. The shape of the rendezvous zone may vary according to the states of the flows.

Two orbits are tangent to each other if the two gyres share an LCS boundary. For a tangent pair *i* and *j*, the agents can communicate with each other, stream data, or exchange information, such as their location, phase, and frequency, as long as they are both within a proximate neighborhood of the tangent point γ_*i*.*j*_ or the *rendezvous zone* Γ_*i,j*_ as shown in Figure 4. The actual range of the rendezvous zone may vary according to the shapes and the states of the flows. For example, the rendezvous zone can be

$$\begin{array}{l} \Gamma_{i.j}=\left\{x\in {\mathbb{R}}^{2}|\|x-\gamma_{i,j}\|<B_{ij}\right\}, \end{array}$$

where *B*_*i,j*_ is a radius pre-selected according to the communication range of both agents *i* and *j*. For the agent traveling along orbit *i*, we mark the phase of the tangential point γ_*i,j*_ as Ψ_*i,j*_, and the phases of it entering and exiting Γ_*i,j*_ as ${\text{Ψ}}_{i,j}^{-}$ and ${\text{Ψ}}_{i,j}^{+}$, with ${\text{Ψ}}_{i,j},{\text{Ψ}}_{i,j}^{-},{\text{Ψ}}_{i,j}^{+}\in \left(-\pi ,\pi \right]$. The rendezvous condition *x*_*i*_ ∈ Γ_*i,j*_∋*x*_*j*_ is therefore equivalent to $\theta_{i}\in \left({\text{Ψ}}_{i,j}^{-},{\text{Ψ}}_{i,j}^{+}\right)\wedge \theta_{j}\in \left({\text{Ψ}}_{j,i}^{-},{\text{Ψ}}_{j,i}^{+}\right)$.

**Figure 5:**
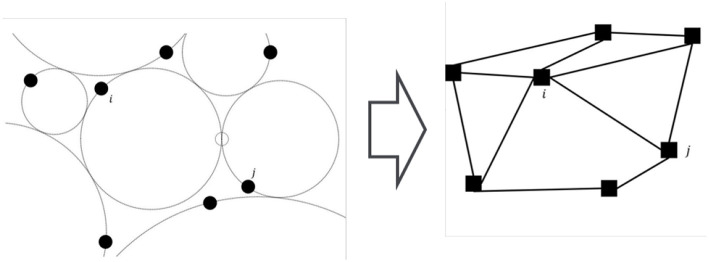
The layout of seven agents on their orbits, and the abstraction to a graph.

Agents are only aware of the existence of other neighboring agents when they are both within the rendezvous zones. We call the *rendezvous duration* the period when a pair of agents are both within the rendezvous zone. Agents can update their control actions, *u*_*i*_, using the exchanged information when they are in the rendezvous zones. Once agents leave the rendezvous zones, they continue executing the same control input which is not updated until the next time they enter the rendezvous zone and exchange information with a neighboring agent. Without loss of generality, we assume *u*_*i*_(*t*) = 0 prior to an agent achieving rendezvous with any neighboring agents for the very first time. For pairs of agents that successfully rendezvous periodically, their *rendezvous period* is denoted by $T_{i,j}$. A pair of agents are *synchronized* if $T_{i,j}$ is shorter than a pre-selected limit. The collection of multiple tangent orbits can be abstract to an undirected graph G=(V,E). Each orbit with an agent is represented as a vertex in $V=\left\{v_{1},\ldots v_{N}\right\}$, and an edge (i,j)∈E if orbits *i* and *j* are tangent to each other. Figure 5 shows an example of how a network of 7 agents is represented as a graph.

In this work, we are interested in the *synchronous rendezvous* of a team of agents deployed on a connected network of orbits. The agents coordinate their motions with neighbors they discovered in the rendezvous zone such that rendezvous occurs periodically and the duration of each rendezvous event is maximized. The problems addressed in this work are listed as follows:

For a team of agents indexed as *i* = 1, …, *N*, each deployed in a circular orbit and together they form a network that can be described as a connected undirected graph G=(V,E). The dynamics of each agent is defined as Equation (1). For any pair of (i,j)∈E, ${\text{Ψ}}_{i,j}^{-},{\text{Ψ}}_{i,j}^{+},{\text{Ψ}}_{j,i}^{-},{\text{Ψ}}_{j,i}^{+}$ are given.

**P1:** For (i,j)∈E, and *u*_*i*_(*t*) = *f*(θ_*i*_(*t*)), *u*_*j*_(*t*) = *f*(θ_*j*_(*t*)) specified for *t* ≥ *t*_0_, we test whether

$$\begin{array}{l} \;\exists t^{rend}>t_{0}\\ \text{s}.\text{t}.\theta_{i}\left(t^{rend}\right)\in \left({\text{Ψ}}_{i,j}^{-},{\text{Ψ}}_{i,j}^{+}\right)\wedge \theta_{j}\left(t^{rend}\right)\in \left({\text{Ψ}}_{j,i}^{-},{\text{Ψ}}_{j,i}^{+}\right). \end{array}$$

If there exists *t*^*rend*^ satisfying the conditions above, we consider agent *i* and *j* are able to rendezvous.

**P2:** We solve for the controller to guarantee further rendezvous

$$\begin{array}{l} \;u_{i}\left(t\right),\forall i=1,\ldots ,N,\forall t>t_{0}\\ \text{s}.\text{t}.\exists t_{ij}^{rend}>t,\forall t>t_{0},\forall i,j\\ \;\theta_{i}\left(t_{ij}^{rend}\right)\in \left({\text{Ψ}}_{i,j}^{-},{\text{Ψ}}_{i,j}^{+}\right)\wedge \theta_{j}\left(t_{ij}^{rend}\right)\in \left({\text{Ψ}}_{j,i}^{-},{\text{Ψ}}_{j,i}^{+}\right). \end{array}$$

For any pair (i,j)∈E, let the dynamics be ${\overset{∙}{\theta }}_{i/j}\left(t\right)=\omega_{i/j}+u_{i/j}\left(t\right)+\eta_{i/j}\left(t\right)$, where η_*i*/*j*_ is a random variable. Let *u*_*i*/*j*_ the same as the solution of **P2**.

**P3.1:** If η_*i,j*_ is a random variable on a bounded interval [−η_*s*_, η_*s*_], we solve for η_*s*_, such that the rendezvous will still be guaranteed to take place.

**P3.2:** If η_*i,j*_ is a Gaussian white noise $N\left(\mu =0,\sigma^{2}\right)$, we solve for *kσ*, such that the rendezvous will still take place with a confidence level of (*P*(*kσ*))^2^.

## 3. Rendezvous Condition Between a Pair of Agents

Whether a pair of oscillators would achieve rendezvous spontaneously has been studied in Wei et al. (2018a,b). In Wei et al. (2018a), oscillators were assumed to be one-dimensional, with at most two rendezvous zones on the left/right end of the line as a proximity around 0 or π. The quest of conditions led to a spontaneous rendezvous between a pair of agents and was addressed through integer programming. Wei et al. (2018b) followed similar methods and derived a decision function *F*(ω_*i*_, ω_*j*_, ϕ_*i*_, ϕ_*j*_) → {1, 0} to determine the possibility of a spontaneous rendezvous based on the initial phases and natural frequencies of both parties.

In this section we analyze the rendezvous condition for a more general case that, by knowing the current phases at *t* = *t*_0_ and future periodic control schemes (for *t* ≥ *t*_0_) for both agents *i* and *j*, whether a further rendezvous would occur or not. As the future motion of both agents is known through θ_*i*/*j*_ = ω_*i*/*j*_ + *u*_*i*/*j*_, the time before either agent's first entrance of the rendezvous zone from now on is denoted as Δ*t*_*i,j*_ (or Δ*t*_*j,i*_) and satisfies

(2) $$\begin{array}{l} \int_{0}^{\Delta t_{i,j}}\omega_{i}+u_{i}\left(t_{0}+t\right)dt={\text{Ψ}}_{i,j}^{-}-\theta_{i}\left(t_{0}\right)\\ \int_{0}^{\Delta t_{j,i}}\omega_{j}+u_{j}\left(t_{0}+t\right)dt={\text{Ψ}}_{j,i}^{-}-\theta_{j}\left(t_{0}\right) \end{array}$$

and the time either agent enters the rendezvous zone for the *m*-th time is $t_{i,j}^{m-}=t_{0}+\Delta t_{i,j}+\left(m-1\right)T_{i}$ (or $t_{j,i}^{m-}=t_{0}+\Delta t_{j,i}+\left(m-1\right)T_{j}$), where *T*_*i*_ and *T*_*j*_ are the periods of both agents. For a pair of agents with no rendezvous before, we take θ_*i*/*j*_(*t*_0_) = ϕ_*i*/*j*_ and *u*_*i*/*j*_ ≡ 0. Then $\Delta t_{i,j/j,i}=\frac{{\text{Ψ}}_{i,j/j,i}^{-}-\phi_{i/j}}{\omega_{i/j}}$, and $t_{i,j/j,i}^{m-}=\Delta t_{i,j/j,i}+\left(m-1\right)\tau_{i/j}$.

The time either agent spends to travel through the rendezvous zone is denoted as δ*t*_*i,j*_ (or δ*t*_*j,i*_) such that

(3) $$\begin{array}{l} \int_{0}^{\delta t_{i,j}}\omega_{i}+u_{i}\left(t_{i,j}^{m-}+t\right)dt={\text{Ψ}}_{i,j}^{+}-{\text{Ψ}}_{i,j}^{-}\\ \int_{0}^{\delta t_{j,i}}\omega_{j}+u_{j}\left(t_{j,i}^{m-}+t\right)dt={\text{Ψ}}_{j,i}^{+}-{\text{Ψ}}_{j,i}^{-}. \end{array}$$

For a pair of agents with no rendezvous before, $\delta t_{i,j/j,i}=\frac{{\text{Ψ}}_{i,j/j,i}^{+}-{\text{Ψ}}_{i,j/j,i}^{-}}{\omega_{i/j}}$.

**Figure 6:**
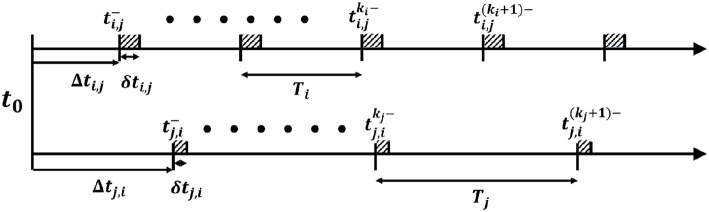
Time schedule for a pair of agents. Shaded parts indicate the time the agents spent in the rendezvous zone. A rendezvous will occur if and only if there is an overlap between shaded parts on both axis.

Therefore the time that agent *i* or *j* is in the rendezvous zone is

(4) $$\begin{array}{l} \underset{m=1,2,\ldots }{⋃}\left(t_{i,j}^{m-},t_{i,j}^{m-}+\delta t_{i,j}\right)\text{ or }\underset{m=1,2,\ldots }{⋃}\left(t_{j,i}^{m-},t_{j,i}^{m-}+\delta t_{j,i}\right) \end{array}$$

respectively. The rendezvous occurs when $\exists t^{rend}>t_{0}$ and *k*_*i*_, *k*_*j*_ ∈ ℕ satisfying the following inequalities (as shown in Figure 6).

(5) $$\begin{array}{l} {\;t}_{i,j}^{k_{i}-}<t^{rend}<t_{i,j}^{k_{i}-}+\delta t_{i,j},\\ \text{and }t_{j,i}^{k_{j}-}<t^{rend}<t_{j,i}^{k_{j}-}+\delta t_{j,i}. \end{array}$$

For Equation (5) to hold, the time set for *i* and *j* to appear in the rendezvous zone has to overlap, which means *i* should enter the rendezvous zone before *j* exists and vice versa, which gives

$$\begin{array}{l} \;\exists \left(k_{i},k_{j}\right)\in {\mathbb{N}}^{2},\text{ s}.\text{t}.\\ \;\Delta t_{i,j}+k_{i}T_{i}<\Delta t_{j,i}+\delta t_{j,i}+k_{j}T_{j},\\ \text{and }\Delta t_{i,j}+\delta t_{i,j}+k_{i}T_{i}>\Delta t_{j,i}+k_{j}T_{j}. \end{array}$$

By rearranging the inequalities we get

Lemma 3.1.* Consider two agents *i* and *j* travel on their corresponding orbits periodically. The time set that agent *i* or *j* appears in a pre-defined rendezvous zone is defined as Equation (4). Then *i* and *j* are able to rendezvous in the future if and only if*

(6) $$\begin{array}{l} \exists \left(k_{i},k_{j}\right)\in {\mathbb{N}}^{2},\;s.t.\\ k_{i}T_{i}-k_{j}T_{j}\in \left(\Delta t_{j,i}-\Delta t_{i,j}-\delta t_{i,j},\;\Delta t_{j,i}-\Delta t_{i,j}+\delta t_{j,i}\right). \end{array}$$

Proof: See Wei et al. (2018a), Corollary 1 and Wei et al. (2018b), Lemma 1.

The solution space S of Equation (6) is a strip bounded by two parallel lines of slope $\frac{T_{i}}{T_{j}}$. Integer solutions are guaranteed to exist as long as S is wider than one unit on either dimension. Therefore, we have

Corollary 3.1.1. *Consider two agents *i* and *j* travel on their corresponding orbits periodically. The time set that agent *i* or *j* appears in a pre-defined rendezvous zone is defined as Equation (4). Then *i* and *j* are able to rendezvous in the future if* $\frac{\delta_{i,j}+\delta_{j,i}}{T_{i}}>1$ *or* $\frac{\delta_{i,j}+\delta_{j,i}}{T_{j}}>1$.

Proof: Corollary 3.1.1 follow directly the proof of Lemma 3.1.

Corollary 3.1.1 is a weak sufficient condition. However, further analysis on Lemma 3.1 reveals much tighter results.

Corollary 3.1.2. *Consider two agents *i* and *j* travel on their corresponding orbits periodically. WLOG, assuming that T*_*i*_ ≤ *T*_*j*_. *The time set that agent *i* or *j* appears in a pre-defined rendezvous zone is defined as Equation (4). Then *i* and *j* will never rendezvous in future iff*.

(7) $$\begin{array}{l} \forall k_{j}\in \mathbb{N},\;\exists k_{i}\in \mathbb{N},\;s.t.\\ k_{i}\in \left(k_{j}\frac{T_{j}}{T_{i}}+\frac{\Delta t_{j,i}-\Delta t_{i,j}+\delta t_{j,i}}{T_{i}}-1,\;k_{j}\frac{T_{j}}{T_{i}}+\frac{\Delta t_{j,i}-\Delta t_{i,j}-\delta t_{i,j}}{T_{i}}\right). \end{array}$$

Proof: Corollary 3.1.2 follows directly the proof of Lemma 3.1.

Notice that Equation (7) holds only when δ*t*_*j,i*_ − *T*_*i*_ < −δ*t*_*i,j*_. If there is δ*t*_*j,i*_ + δ*t*_*i,j*_ ≥ *T*_*i*_, Equation (7) cannot hold and *i* and *j will* rendezvous. This echos Corollary 3.1.1.

We now analyze Equation (7) in two categories: (i) $\frac{T_{i}}{T_{j}}$ is a rational number, and $\frac{T_{i}}{T_{j}}$ can be reduced to $\frac{I_{i}}{I_{j}}$ with $I_{i},I_{j}\in {\mathbb{Z}}^{+}$ are co-prime; and (ii) $\frac{T_{i}}{T_{j}}$ is an irrational number. For the first case,

Lemma 3.2. *Consider two agents *i* and *j* travel on their corresponding orbits periodically. The time set that agent *i* or *j* appears in a pre-defined rendezvous zone is defined as Equation (4), with* $\frac{T_{i}}{T_{j}}=\frac{I_{i}}{I_{j}}$, and $\frac{I_{i}}{I_{j}}$ *is a proper irreducible fraction. Then *i* and *j* are guaranteed to rendezvous in the future if the set*

$$\begin{array}{l} \{(I_{i},I_{j})\in {\mathbb{R}}^{2}|I_{i}T_{i}-(I_{j}+1)T_{j}+\Delta t_{i,j}-\Delta t_{j,i}+\delta t_{i,j}<0\wedge I_{i}T_{i}\\ \;-I_{j}T_{j}+\Delta t_{i,j}-\Delta t_{j,i}-\delta t_{j,i}>0\} \end{array}$$

*has a non-empty intersection with* ℕ ^2^

Proof: Follow the steps for (Wei et al., 2018a, Corollary 1).

Notice that Lemma 3.2 suggests a similar but stronger result as in (Wei et al., 2018a, Proposition 2), which is derived following *Khinchine's flatness theorem* (as in Dadush, 2012).

Lemma 3.2 suggests that after a time period of length **T** = *I*_*j*_*T*_*i*_ = *I*_*i*_*T*_*j*_, both agents would have completed integer multiples of rounds and appears at the same locations of *t*_0_. The rendezvous must happen before this *t*_0_ + **T** or a future rendezvous will never occur. The test of whether a pair of agents are able to discover each other (e.g., rendezvous under an unforced fashion for the first time) can be tested within a finite time window. For any *t*_*rend*_ that both θ_*i*_(*t*_*rend*_) and θ_*j*_(*t*_*rend*_) are inside the rendezvous zone, θ_*i*_(*t*_*rend*_ + **T**) and θ_*j*_(*t*_*rend*_ + **T**) will be as well. Thus, *i* and *j* are synchronized directly, and **T** can be seen as a conservative estimate of the rendezvous period of *i* and *j*, or to say $T_{i,j}\leq \text{T}$.

When $\frac{T_{i}}{T_{j}}$ is irrational, there will not be a periodic pattern in the agents' performance, and therefore we can neither test for a future rendezvous within a finite time nor guarantee a synchronized recurrence of rendezvous. On the other hand, some other results can be derived, saying that *i* and *j* always rendezvous in this case.

Lemma 3.3. *Consider two agents *i* and *j* travel on their corresponding orbits periodically. The time set that agent *i* or *j* appears in a pre-defined rendezvous zone is defined as Equation (4), with* $\frac{T_{i}}{T_{j}}$ *an irrational number, and at least one of δ*t*_*i,j*_ and δ*t*_*j,i*_ is non-zero. Then *i* and *j* rendezvous infinitely often*.

Proof: See Appendix.

Together with previous lemmas, the cases that agents *i* and *j* never rendezvous is significantly narrowed to a rational ratio of *T*_*i*_ and *T*_*j*_ with some certain combinations of rendezvous zones and current phases.

Theorem 3.4. *Consider two agents *i* and *j* travel on their corresponding orbits periodically. WLOG, assuming *T*_*i*_ ≤ *T*_*j*_. The time set that agent *i* or *j* appears in a pre-defined rendezvous zone is defined as Equation (4). Then *i* and *j* are not able to rendezvous if and only if (i) $\frac{T_{i}}{T_{j}}$ is a rational number; and (ii) the condition in Equation (7) holds for some k_i_* ∈ 1, …, *I_j_ and k_j_* ∈ 1, …, *I_i_*.

Corollary 3.4.1. *Consider two agents *i* and *j* travel on their corresponding orbits periodically. The time set that agent *i* or *j* appears in a pre-defined rendezvous zone is defined as Equation (4). Then *i* and *j* are guaranteed to rendezvous if (i) $\frac{T_{i}}{T_{j}}$ is an irrational number; or (ii) $\delta t_{i,j}+\delta t_{j,i}>\frac{T_{j}}{I_{j}}$*.

Proof: Theorem 3.4 and its corollary follow directly the proof of previous lemmas and corollaries.

Since the set of rational numbers has a Lebesgue measure of zero, we consider it is almost impossible for a pair of agents to maintain frequencies with an exact rational ratio. Practically, for agents actuated to maintain such frequencies intentionally, noises and disturbance always arise to deviate the agents and result in (most likely) irrational ratios. According to our analysis, any tangent pair of agents are almost always able to discover each other and are re-united in the rendezvous region. The sensitivity analysis is provided in section 5.

## 4. Synchronous Rendezvous and Design of Controllers

### 4.1. Synchronizing a Pair of Agents

Although a pair of agents are able to reach rendezvous relying solely on their frequencies holding an irrational ratio, such rendezvous cannot happen periodically, and the next rendezvous may not occur until after a long interval. To synchronize a pair into periodic rendezvous, the agents' motion will be actuated to yield desired frequencies. For agents *i* and *j* with a common rendezvous zone Γ_*i,j*_, ideally, either agent shall travel at a constant angular velocity outside of the rendezvous zone, such that it is able to return to the zone after completing integer multiples of periods *T*_*i*/*j*_. A periodic rendezvous occurs only when $\frac{T_{i}}{T_{j}}$ is a rational number, and if *i* completes *m*_*i*_ periods in approximately the same amount of time of *j* completing *m*_*j*_ periods, where *m*_*i*_, *m*_*j*_ ∈ ℕ. The rendezvous period $T_{i,j}$ is therefore a common multiple of *T*_*i*_ and *T*_*j*_ that satisfies $T_{i,j}=m_{i}T_{i}=m_{j}T_{j}$, apparently, $\frac{m_{j}}{m_{i}}=\frac{T_{i}}{T_{j}}$. If $T_{ij}$ is shorter than a finite time limit, we consider this pair of agents reaches a synchronous rendezvous.

When the pair are both in the rendezvous zone, a coordinating controller can be applied to actuate them such that a new pair of angular velocities with a desired rational ratio will be reached and maintained before either party exiting the rendezvous zone. Meanwhile, this controller is tasked with maximizing the rendezvous duration, which can be realized by regulating their motions to align them before exiting the rendezvous zone. In this section we show an example of designing a time-optimal controller to adjust both agents' angular velocities to the mean value, and align the agents to hit the tangent point at the same time, which satisfies

$$\begin{array}{l} \theta_{i}\left(t\right)-\theta_{j}\left(t\right)={\text{Ψ}}_{i,j}-{\text{Ψ}}_{j,i}+2k\pi ,\;k\in \mathbb{Z}. \end{array}$$

The synchronization task is accomplished in a split way. WLOG agent *j* adjusts its frequency to the desired value, and the other agent takes *j* as an anchor and adjusts its own frequency and phase to track agent *j*. In this case, the desired frequency is the mean of the pair's angular velocity before the rendezvous was initiated. This new frequency shall be achieved by agent *j* through applying

(8) $$\begin{array}{l} u_{j}\left(t\right)=\frac{{\overset{∙}{\theta }}_{i}\left(t^{rend}\right)+{\overset{∙}{\theta }}_{j}\left(t^{rend}\right)}{2}-{\overset{∙}{\theta }}_{j}\left(t^{rend}\right). \end{array}$$

The controller letting agent *i* track *j* is set to be time-optimal to give the pair the best chance to accomplish the synchronization task within a very limited rendezvous duration. The controller can be implemented by defining the error states as

$$\begin{array}{l} \epsilon_{1}=\theta_{i}\left(t\right)-\theta_{j}\left(t\right)-\left\lfloor \frac{\theta_{i}\left(t\right)-\theta_{j}\left(t\right)}{2\pi }\right\rfloor 2\pi -\left(\psi_{i,j}-\psi_{j,i}\right),\\ \epsilon_{2}={\overset{∙}{\theta }}_{i}\left(t\right)-{\overset{∙}{\theta }}_{j}\left(t\right). \end{array}$$

with the error dynamics as follows

(9) $$\begin{array}{l} \left[\begin{array}{c} {\overset{∙}{\epsilon }}_{1}\\ {\overset{∙}{\epsilon }}_{2} \end{array}\right]=\left[\begin{array}{cc} 0 & 1\\ 0 & 0 \end{array}\right]\left[\begin{array}{c} \epsilon_{1}\\ \epsilon_{2} \end{array}\right]+\left[\begin{array}{c} 0\\ v_{i,j} \end{array}\right]. \end{array}$$

The control input in Equation (9), *v*_*i,j*_, is the difference between *u*_*i*_ and *u*_*j*_. As *u*_*j*_ is determined by Equation (8), *u*_*i*_ can be acquired straightforwardly by designing a bang-bang controller following Athans and Falb (2007). The details are hereby omitted. Notice that the controllers here are designed to be time optimal, and we set the desired frequencies as the mean value of both agents as an example. It is also possible to design controllers with other optimization objectives, such as minimizing the energy required.

### 4.2. Synchronizing Multiple Agents in a Network

After rendezvous was initiated between a pair of agents, they synchronize themselves to a common rendezvous period that should become invariant. Such synchronization can be extended to a connected network of multiple agents. Wei et al. (2018a) proposed one way that could be used in a chain of one-dimensional oscillators, such that any pair of agents would have only one chance of actuation. A pair of agents that have never actuated their frequencies would both be synchronized to the average angular velocity of theirs while reaching rendezvous and be locked to this *committed* frequency. When any of the committed agents reaches rendezvous simultaneously with an uncommitted agent, the uncommitted one would join this committed frequency. This protocol can be extended to higher dimensional agents that form chains or trees. It was also shown in Wei et al. (2018a) that the implementation of this protocol may lead to the creation of sub-graphs of synchronized agents, with each sub-graph having its own committed frequency.

Wei et al. (2018b) suggests an alternative synchronization policy which can be implemented if all agents are allowed to modify their frequencies on a continuous basis. Interaction between agents is still bounded to the intermittent and brief rendezvous duration, but every pair of agents will switch to their average angular velocity while in rendezvous,

(10) $$\begin{array}{l} {\overset{∙}{\theta }}_{i}\left(t^{+}\right)={\overset{∙}{\theta }}_{j}\left(t^{+}\right)=\frac{1}{2}\left({\overset{∙}{\theta }}_{i}\left(t^{rend}\right)+{\overset{∙}{\theta }}_{j}\left(t^{rend}\right)\right). \end{array}$$

Wei et al. (2018b) shows that the frequency synchronization propagates to the whole network if all agents are connected intermittently, and eventually the agents converge to a common oscillating frequency:

Theorem 4.1. *For *N* agents connected over a graph, each having an initial frequency* ${\overset{∙}{\theta }}_{i}\left(0\right)=\omega_{i}$ *for i* = 1, …, *N*, *and assuming that the condition of Theorem 3.4 is always false for any pair of adjacent agents, then all agents' frequencies converge to the average of their initial values, i.e., for all i* ∈ {1, …, *N*}, $\underset{t\to \infty }{\lim}{\dot{\theta }}_{i}\left(t\right)={\bar{\omega }}_{N}=\frac{1}{N}\overset{N}{\underset{n=1}{\sum }}\omega_{n}$.

Proof: See Appendix.

The discussion in section 3 points out it is extremely rare for a neighboring pair of agents *not* to rendezvous in practice. In the even rarer cases that certain links in the graph are sabotaged due to a loss of rendezvous occurring (e.g., all pairs forming theses links meet the very rare condition of Theorem 3.4,) and the graph becomes disconnected, the network shall exhibit several isolated components, with each isolated component achieving its own synchronization.

Section 3 also points out that one key factor to make sure a pair of agents always rendezvous in the future is that noise and disturbance exist in the system which deviate the frequency ratio to a most likely irrational number. It is also worth noticing that, for an already synchronized pair of agents, the achieved (rational) frequency ratio may also be deviated by the inevitable noise and disturbance. The resulting irrational ratio may not cause a total loss of future rendezvous, but is still able to result in a much longer rendezvous period that is not applicable for certain real world cases. In section 5 we will analyze the effect of noise and disturbance.

## 5. Sensitivity Analysis

The analysis in section 3 provides a theoretical basis for a promised future rendezvous for any pair of agents on neighboring circular orbits. However, in practice, there are at least two types of factors that may cause a loss of a synchronized rendezvous scheme: (i) There is usually an upper bound of the rendezvous period due to the requirements of the specific application, for example (but not limited to) the recharging of an agent, uploading data from an agent's limited storage, or a regular recalibration of an agent; and (ii) the disturbance and noise that accumulated in the agents' dynamics, especially when an agent is not in rendezvous, which deviate the agents' angular velocities from the desired value.

Definition 1. If agents *i* and *j* are synchronized to a periodic rendezvous that occurs every $T_{i,j}=m_{i}T_{i}=m_{j}T_{j}$ amount of time, with *m*_*i*_.*m*_*j*_ ∈ ℕ, and *m*_*i*_, *m*_*j*_ co-prime, we say that agents *i* and *j* are synchronized to an *m*_*i*_-*m*_*j*_ rendezvous scheme. A synchronized pair fails to maintain its *m*_*i*_-*m*_*j*_ rendezvous scheme is said to be *desynchronized*.

In this section we analyze in what conditions the effect of the disturbance and noise will cause a synchronized pair of agents fail to rendezvous in their pre-selected scheme and, when such desynchronization happens, whether they would be able to synchronize themselves into another periodic rendezvous scheme. Consider agents *i* and *j* on a pre-selected *m*_*i*_-*m*_*j*_ scheme, where WLOG *T*_*i*_ < *T*_*j*_, and $T_{i,j}<\bar{T}$, $\bar{T}$ is the required upper bound of the rendezvous period. Between two consecutive rendezvous, the control inputs remain constant, such that

(11) $$\begin{array}{l} \left(\omega_{i/j}+u_{i/j}\right)T_{i,j}=2\pi . \end{array}$$

The agent dynamics contains certain noise that results in a disturbance on the control input. The dynamics shown in Equation (1) is therefore rewritten as

(12) $$\begin{array}{l} {\overset{∙}{\theta }}_{i/j}\left(t\right)=\omega_{i/j}+\left(u_{i/j}\left(t\right)+\eta_{i/j}\right), \end{array}$$

where η_*i*/*j*_ is the disturbance associated with agent *i* and *j*. Either of η_*i*/*j*_ is an independent random variable. The actual period of either agent, ${\tilde{T}}_{i/j}$ satisfies

(13) $$\begin{array}{l} \omega_{i/j}{\tilde{T}}_{i/j}+\int_{0}^{{\tilde{T}}_{i/j}}\left(u_{i/j}\left(t\right)+\eta_{i/j}\right)dt=2\pi . \end{array}$$

We discuss two typical types of η, (i) that η_*i*/*j*_ is a random variable on a bounded interval [−η_*s*_, η_*s*_]; and (ii) that η_*i*/*j*_ is unbounded, but a Gaussian white noise with a mean of μ = 0 and a standard deviation σ. In the first case that η is bounded, we have ${\tilde{T}}_{i}$ bounded by ${\tilde{T}}_{i}\in \left[\frac{2\pi }{\omega_{i}+u_{i}+\eta_{s}},\frac{2\pi }{\omega_{i}+u_{i}-\eta_{s}}\right]$. Together with Equation (11), we have

(14) $$\begin{array}{l} T_{i}-\frac{\eta_{s}T_{i}}{\omega_{i}+u_{i}+\eta_{s}}\leq {\tilde{T}}_{i}\leq T_{i}+\frac{\eta_{s}T_{i}}{\omega_{i}+u_{i}-\eta_{s}}, \end{array}$$

and ${\tilde{T}}_{j}$ bounded the same way.

Let $\alpha_{i,j}=\frac{{\tilde{T}}_{i}}{{\tilde{T}}_{j}}$ denote the portion that *j* completes α_*i,j*_ of a circle while *i* completes one circle. According to Equation (14), α_*i,j*_ is bounded by

(15) $$\begin{array}{l} \frac{\omega_{j}+u_{j}-\eta_{s}}{\omega_{i}+u_{i}+\eta_{s}}\leq \alpha_{i,j}\leq \frac{\omega_{j}+u_{j}+\eta_{s}}{\omega_{i}+u_{i}-\eta_{s}}. \end{array}$$

Take the time that *i* exits the rendezvous zone as the beginning of the next period, and let the time that *j* exits the rendezvous zone as $\Delta {\tilde{t}}_{j,i}\in \left(-\frac{\delta t_{i,j}}{T_{i}}{\tilde{T}}_{i},\frac{\delta t_{j,i}}{T_{j}}{\tilde{T}}_{j}\right)$. The time window that *i* is able to rendezvous with *j* while completing its *m*_*i*_-th circle is $\left[\left(m_{i}-\frac{\delta t_{i,j}}{T_{i}}\right){\tilde{T}}_{i},m_{i}{\tilde{T}}_{i}\right]$, and *j* is able to rendezvous with *i* while completing its *m*_*j*_-th circle is $\left[\left(m_{j}-\frac{\delta t_{j,i}}{T_{j}}\right){\tilde{T}}_{j}+\Delta {\tilde{t}}_{j,i},m_{j}{\tilde{T}}_{j}+\Delta {\tilde{t}}_{j,i}\right]$.

Lemma 5.1. *For a pair of tangent agents with dynamics defined as in (12), and η is a random variable on a bounded interval* [*−η_*s*_, η_*s*_*]*. A controller is designed following section 4 to synchronize them to a pair of desired periods *T*_*i*_ and *T*_*j*_, such that *m*_*i*_*T*_*i*_ = *m*_*j*_*T*_*j*_, with *m*_*i*_, *m*_*j*_* ∈ ℕ. *The pair is guaranteed to rendezvous on an *m*_*i*_-*m*_*j*_ scheme under the effect of disturbance iff*. $\exists \Delta {\tilde{t}}_{j,i}\in \left(-\frac{\delta t_{i,j}}{T_{i}}{\tilde{T}}_{i},\frac{\delta t_{j,i}}{T_{j}}{\tilde{T}}_{j}\right)$, *such that* $\forall \alpha_{i,j}=\frac{{\tilde{T}}_{i}}{{\tilde{T}}_{j}}\in \left[\frac{\omega_{j}+u_{j}-\eta_{s}}{\omega_{i}+u_{i}+\eta_{s}},\frac{\omega_{j}+u_{j}+\eta_{s}}{\omega_{i}+u_{i}-\eta_{s}}\right]$, *the following inequalities are satisfied*.

(16) $$\begin{array}{l} \;m_{i}{\tilde{T}}_{i}>\left(m_{j}-\frac{\delta t_{j,i}}{T_{j}}\right){\tilde{T}}_{j}+\Delta {\tilde{t}}_{j,i},\\ and\;m_{j}{\tilde{T}}_{j}+\Delta {\tilde{t}}_{j,i}>\left(m_{i}-\frac{\delta t_{i,j}}{T_{i}}\right){\tilde{T}}_{i}. \end{array}$$

Proof: Given the agents are aligned such that $\Delta {\tilde{t}}_{j,i}$ satisfies (16), there is always an overlap of agents *i* and *j*'s time in the rendezvous zone. While both agents find themselves in the rendezvous zone, they are able to actuate themselves and re-align their phases to satisfy (16), expecting the next rendezvous to occur. The pair therefore conducts a rendezvous on an *m*_*i*_-*m*_*j*_ periodic scheme.

Since in practice, both $\frac{\delta t_{j,i}}{T_{j}}$ and $\frac{\delta t_{i,j}}{T_{i}}$ are infinitesimal, sometimes we may take $\frac{\delta t_{j,i}}{T_{j}}=\frac{\delta t_{i,j}}{T_{i}}$ here, which yields the following result.

Theorem 5.2. *For a pair of tangent agents with dynamics defined as in (12), and η is a random variable on a bounded interval* [*−η_*s*_, η_*s*_*]*. A controller is designed following section 4 to synchronize them to a pair of desired periods *T*_*i*_ and *T*_*j*_, such that *m*_*i*_*T*_*i*_ = *m*_*j*_*T*_*j*_, with *m*_*i*_, *m*_*j*_* ∈ ℕ. *The pair is guaranteed to rendezvous on an *m*_*i*_-*m*_*j*_ scheme under the effect of disturbance if and only if*

(17) $$\begin{array}{l} \eta_{s}<\frac{\omega_{i}+u_{i}}{m_{i}}\left(\frac{m_{i}\frac{\delta t_{j,i}}{T_{j}}+m_{j}\frac{\delta t_{i,j}}{T_{i}}}{m_{i}+m_{j}+|\frac{\delta t_{i,j}}{T_{i}}-\frac{\delta t_{j,i}}{T_{j}}|}\right). \end{array}$$

If the rendezvous zone is defined such $\frac{\delta t_{j,i}}{T_{j}}=\frac{\delta t_{i,j}}{T_{i}}$, then

(18) $$\begin{array}{l} \eta_{s}<\frac{\delta t_{i,j}\left(\omega_{i}+u_{i}\right)}{m_{i}T_{i}}. \end{array}$$

Proof: See Appendix.

For disturbance greater than the limit provided in Corollary 5.2, agents are not able to rendezvous in the pre-selected *m*_*i*_-*m*_*j*_ scheme. However, it is still possible that the pair may fall into another scheme. We denote $\lambda_{i}=\frac{{\text{Ψ}}_{i,j}^{+}-{\text{Ψ}}_{i,j}^{-}}{2\pi }$, and $\lambda_{j}=\frac{{\text{Ψ}}_{j,i}^{+}-{\text{Ψ}}_{j,i}^{-}}{2\pi }$, the following result can be derived.

Lemma 5.3. *For a pair of tangent agents with dynamics defined as in (12), as ${\tilde{T}}_{i}$ and ${\tilde{T}}_{j}$ determined through (13), and $\alpha_{i,j}=\frac{{\tilde{T}}_{i}}{{\tilde{T}}_{j}}$, the pair is guaranteed to rendezvous on an *m*_*i*_-*m*_*j*_ scheme if and only if*

(19) $$\begin{array}{l} \alpha_{i,j}\in \left(\frac{m_{j}-\lambda_{j}}{m_{i}},\frac{m_{j}}{m_{i}-\lambda_{i}}\right). \end{array}$$

Proof: Lemma 5.3 follow directly the proof of Lemma 5.1.

If the requirements of the specific application arise that the pair needs to rendezvous before $\bar{T}$ amount of time, if $\bar{T}\geq {\tilde{T}}_{j}$, the condition becomes

Lemma 5.4. *For a pair of tangent agents with dynamics defined as in (12). As ${\tilde{T}}_{i}$ and ${\tilde{T}}_{j}$ determined through (13), and $\alpha_{i,j}=\frac{{\tilde{T}}_{i}}{{\tilde{T}}_{j}}$, the pair is guaranteed to rendezvous before agent *j* completes *M*_*j*_ periods if and only if*

(20) $$\begin{array}{l} \alpha_{i,j}\in \overset{M_{j}}{\underset{m_{j}=1}{⋃}}\underset{\forall m_{i}\in \mathbb{N}}{⋃}\left(\frac{m_{j}-\lambda_{j}}{m_{i}},\frac{m_{j}}{m_{i}-\lambda_{i}}\right). \end{array}$$

Proof: This lemma holds directly following Lemma 5.3.

For any given pair of *m*_*i*_ and *m*_*j*_, the range of a valid α_*i,j*_ is a neighborhood around $\frac{m_{j}}{m_{i}}$. For a fixed *m*_*j*_, with the increase of *m*_*i*_, the distribution of $\frac{m_{j}}{m_{i}}$ becomes more and more dense. When *m*_*i*_ is great enough, all valid ranges of α_*i,j*_ overlap with each other and form a continuous range.

Theorem 5.5. *For a pair of tangent agents with dynamics defined as in (12). As ${\tilde{T}}_{i}$ and ${\tilde{T}}_{j}$ determined through (13), and $\alpha_{i,j}=\frac{{\tilde{T}}_{i}}{{\tilde{T}}_{j}}$, the pair is guaranteed to rendezvous within $\bar{T}<\infty$ amount of time if*

(21) $$\begin{array}{l} {\tilde{T}}_{j}\leq \bar{T}\;and\;\alpha_{i,j}<\frac{1}{M_{i}-\lambda_{i}},\\ \;where\;M_{i}=\lceil \frac{1-\lambda_{i}-\lambda_{j}+\lambda_{i}\lambda_{j}}{\lambda_{j}}\rceil . \end{array}$$

Proof: See Appendix.

Corollary 5.5.1. *For a pair of tangent agents with dynamics defined as in (12), and η is a random variable on a bounded interval* [*−η_*s*_, η_*s*_*]*, a controller is designed following section 4 to synchronize them to a pair of desired periods *T*_*i*_ and *T*_*j*_, such that *m*_*i*_*T*_*i*_ = *m*_*j*_*T*_*j*_, with *m*_*i*_, *m*_*j*_* ∈ ℕ. *The pair is guaranteed to rendezvous under the effect of disturbance if*

$$\begin{array}{l} \eta_{s}<\frac{\bar{T}\left(\omega_{j}+u_{j}\right)-2\pi }{\bar{T}}\;and\;\eta_{s}<\frac{\left(\omega_{i}+u_{i}\right)-\left(M_{i}-\lambda_{i}\right)\left(\omega_{j}+u_{j}\right)}{M_{i}-\lambda_{i}+1}\\ \;where\;M_{i}=\lceil \frac{1-\lambda_{i}-\lambda_{j}+\lambda_{i}\lambda_{j}}{\lambda_{j}}\rceil . \end{array}$$

Notice that Theorem 5.5 and Corollary 5.5.1 suggest that there exists some gap between the valid ranges of α_*i,j*_, such that for *m*_*j*_ ≤ *M*_*j*_, *i* and *j* are not guaranteed for a future rendezvous. It is because the set of all rational numbers $\frac{m_{j}}{m_{i}}$ with a finite *m*_*j*_ is *not* dense on the real number axis. If *m*_*j*_ can go infinitely large such that all rational numbers are included, the gaps are narrowed to only some points upon certain rational numbers.

For the case that η is not bounded but a Gaussian white noise $N\left(\mu =0,\sigma^{2}\right)$, the actual distribution of $\alpha_{i,j}=\frac{\omega_{j}+u_{j}+\eta_{i}}{\omega_{i}+u_{i}+\eta_{j}}$ is a Cauchy distribution with both tails determined case by case through the specific values of ω_*i*_ + *u*_*i*_ and ω_*j*_ + *u*_*j*_. As a necessary and sufficient condition on η that yields certain rendezvous schemes with a good confidence is hard to solve analytically, a sufficient condition is still relatively easy to obtain. The probability that a normal deviate lies in the range between (μ − *kσ*, μ + *kσ*) is given by

$$\begin{array}{l} P\left(k\sigma \right)=F\left(\mu +k\sigma \right)-F\left(\mu -k\sigma \right)=\text{erf}\left(\frac{k}{\sqrt{2}}\right), \end{array}$$

where *F* is the cumulative distribution function.

The probability of α_*i,j*_ falls between the bounds

$$\begin{array}{l} \alpha_{i,j}\in \left(\frac{\omega_{j}+u_{j}-k\sigma }{\omega_{i}+u_{i}+k\sigma },\frac{\omega_{j}+u_{j}+k\sigma }{\omega_{i}+u_{i}-k\sigma }\right) \end{array}$$

is simply (*P*(*kσ*))^2^. Thus we have

Theorem 5.6. *For a pair of tangent agents with dynamics defined as in (12), and each of η_*i,j*_ is $N\left(\mu =0,\sigma^{2}\right)$. A controller is designed following section 4 to synchronize them to a pair of desired periods *T*_*i*_ and *T*_*j*_, such that *m*_*i*_*T*_*i*_ = *m*_*j*_*T*_*j*_, with *m*_*i*_, *m*_*j*_* ∈ ℕ. *If the rendezvous zone is defined such that $\frac{\delta t_{j,i}}{T_{j}}=\frac{\delta t_{i,j}}{T_{i}}$ holds, the pair will rendezvous on an *m*_*i*_-*m*_*j*_ scheme with a confidence level of at least* (*P*(*kσ*))^2^ *under the effect of disturbance if*

(22) $$\begin{array}{l} k\sigma <\frac{\delta t_{i,j}\left(\omega_{i}+u_{i}\right)}{2m_{i}T_{i}-\delta t_{i,j}}. \end{array}$$

Theorem 5.7. *For a pair of tangent agents with dynamics defined as in (12), and each of η_*i,j*_ is $N\left(\mu =0,\sigma^{2}\right)$. A controller is designed following section 4 to synchronize them to a pair of desired periods *T*_*i*_ and *T*_*j*_, such that *m*_*i*_*T*_*i*_ = *m*_*j*_*T*_*j*_, with *m*_*i*_, *m*_*j*_* ∈ ℕ. *will rendezvous on a periodic scheme with a confidence level of at least* (*P*(*kσ*))^2^ *under the effect of disturbance if*

$$\begin{array}{l} \;\left(M_{i}-\lambda_{i}\right)\left(\omega_{j}+u_{j}+k\sigma \right)<\omega_{i}+u_{i}-k\sigma ,\\ where\;M_{i}=\lceil \frac{1-\lambda_{i}-\lambda_{j}+\lambda_{i}\lambda_{j}}{\lambda_{j}}\rceil . \end{array}$$

Proof: Both theorems follow Theorem 5.2 and Corollary 5.5.1 directly.

## 6. Simulation

**Figure 7:**
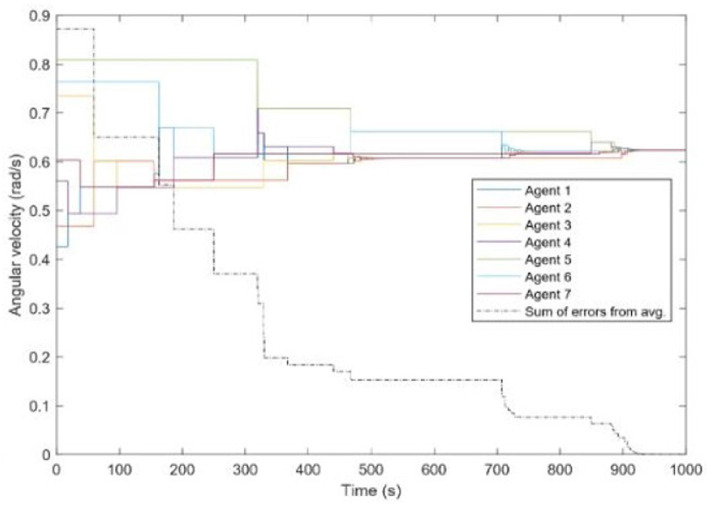
Seven agents converge to the same frequency; the black dash line shows how Δ(*t*) decreases.

In this section we show simulations of the synchronization and desynchronization of a network of multiple agents. We first show seven agents deployed as shown in Figure 5 synchronized without existence of noise or disturbance. The initial phases of the agents are randomly generated, and the natural frequencies of all agents were randomly selected between [0.1π, 0.25π]. Any agent would only know about the others after rendezvous with them simultaneously. Figure 7 shows the synchronization of their frequencies. The dash line shows Δ(*t*). The system is synchronized while Δ(*t*) decreased to zero.

**Figure 8:**
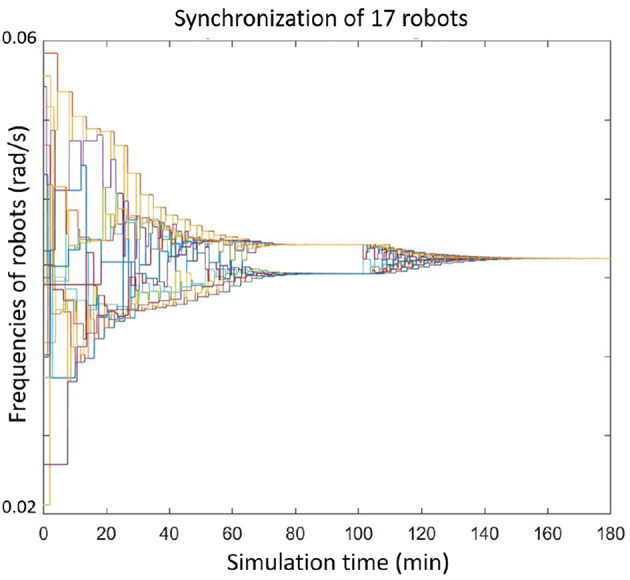
Seventeen agents converge to the same frequency.

**Figure 9:**
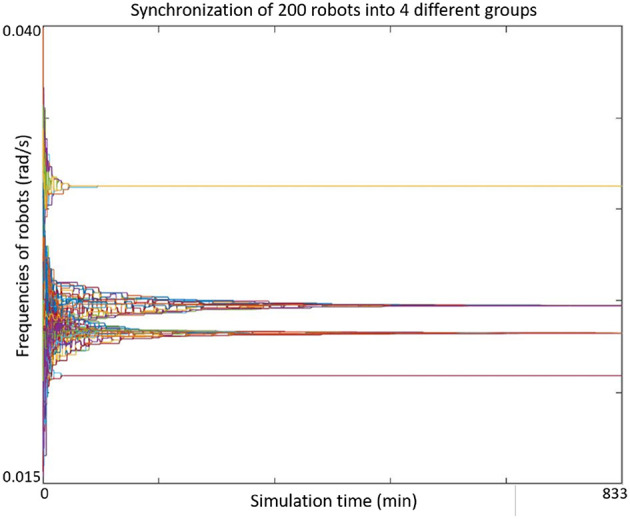
Two-hundred agents converge to four subgroups.

Figures 8, 9 show more examples of synchronizing 17 or 200 agents. Notice that in Figure 9 the network of the agents were not connected, but separated into four sub-graphs.

**Figure 10:**
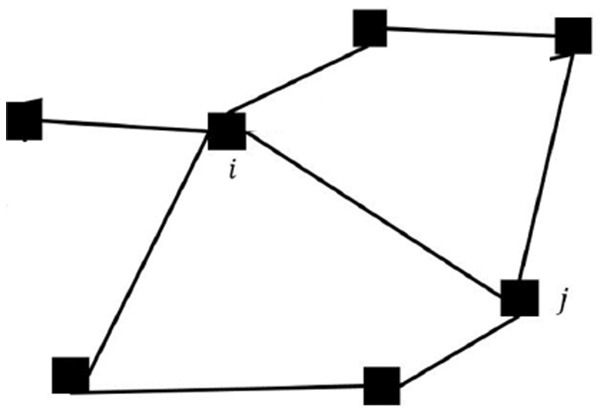
The rendezvous network formed by the agents synchronized following the second approach. An edge means that the pair is able to rendezvous periodically with a period no longer than $\bar{T}=30$ s.

**Figure 11:**
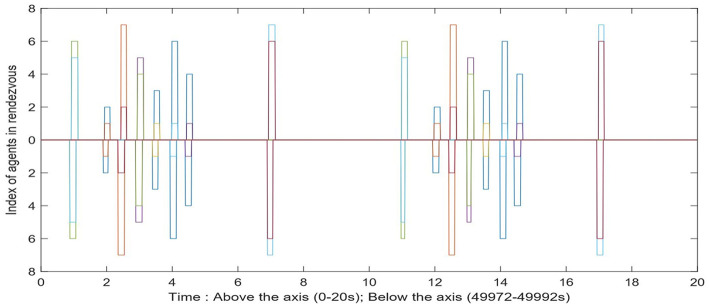
The comparison between the rendezvous events in the beginning and near the end of the simulation. Every bar has two readings, indicating that the two agents are in rendezvous at this time. The width of each bar is the rendezvous duration.

Now we show the effect of the noise and disturbance on the synchronized system. We take the synchronized groups formed by seven agents, which is shown as in Figure 10. We introduce white noise to all agents' dynamics. The noise follows a normal distribution with zero mean and different levels of σ. A pair of agents are considered to be *synchronized* with the existence of noise only if they are able to rendezvous every $\bar{T}=30s$. By choosing 3σ = η_*s*_, the network almost always maintains its current configuration. We ran the simulation for a 50,000 s time window. Figure 11 shows a comparison between the rendezvous events in the very beginning (0–20 s) and close to the end (49, 972–49, 992 s) of the simulation. The distribution of the rendezvous events are almost identical in both time periods. Only some of the rendezvous events are shorter due to the accumulated disturbance delaying one of them from joining the rendezvous as scheduled.

While the disturbance is set to be a normal distribution of zero mean, and 2σ = η_*s*_, the network is more likely to be desynchronized.

**Figure 12:**
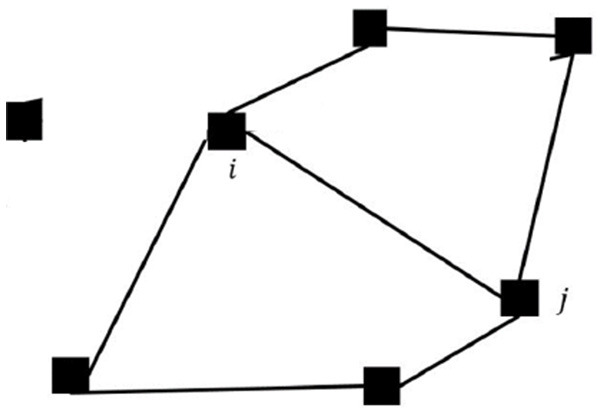
The rendezvous network after this group of agents has been desynchronized due to the existence of a disturbance. Agent 3 is disconnected from all the neighbors, and the network was split into two sub-graphs.

**Figure 13:**
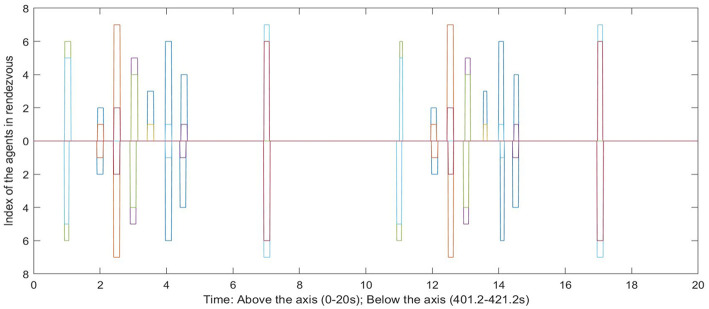
The comparison between the rendezvous events in the beginning and near the end of the simulation. The bar indicating the rendezvous between agent 1 and 3 disappeared below the axis (after 400 s of simulation).

**Figure 14:**
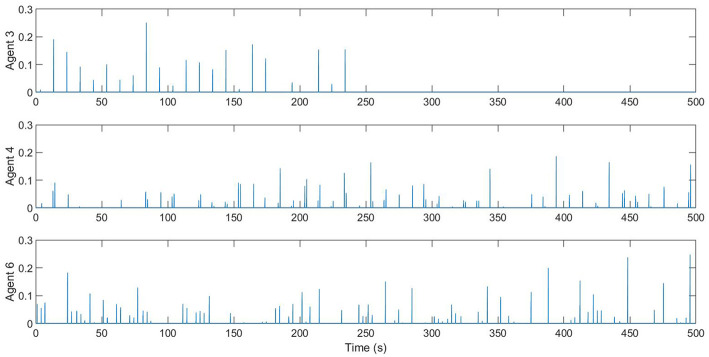
The control input to align an agent's phases with any companion it is in rendezvous with. Agent 3 failed to rendezvous with any of its neighbors since ~ 240 s, and therefore no control input was generated after that time.

Figure 12 shows an example that the network is disconnected under this level of disturbance. Figure 13 is the comparison between the rendezvous events on the first 20 s and after 400 s. We can see that the short bar indicating the rendezvous between 1 and 3 disappeared. Figure 14 shows the control input on Agents 3, 4, and 6 to align them with their companions in rendezvous. Notice that the controllers are only activated while an agent is in rendezvous. We can see that Agent 4 and 6 have rendezvous events all the time, but Agent 3 has no rendezvous event with any other agent after ~ 240 s.

## 7. Conclusion

This paper addressed a synchronous rendezvous problem for a network of mobile sensors monitoring large-scale ocean regions bounded by LCS. It approximated the coherent structures as circular orbits tangential to each other, and assuming that the agents flowing along these orbits can only interact while in close proximity, this formulation gave rise to a graph of intermittently interacting 2-D oscillators. Conditions under which a pair of oscillators can rendezvous solely relying on flow dynamics were presented, controllers were designed to lock them into subsequent periodic rendezvous, and sensitivity analysis was provided for two typical types of disturbance on the control input.

The results in this paper can also find use in other fields, such as perimeter surveillance or space docking. In this work, agents are assumed to travel only along their own circular orbits. Future directions include allowing agents to drive off the orbits to explore the inner circle of the bounded region and to optimally plan its trajectory subject to the ocean environment.

## Author Contributions

XY and CW developed the theoretical formalism under supervision of MH and HT. XY performed the simulation under supervision of MH. XY and MH wrote the manuscript with support from CW and HT.

### Conflict of Interest Statement

The authors declare that the research was conducted in the absence of any commercial or financial relationships that could be construed as a potential conflict of interest.

## Footnotes

^1^Noise can arise from uncertainty in model parameters and/or measurement noise.

^2^For the full animation visit http://svs.gsfc.nasa.gov/vis/a000000/a003800/a003827/

## Appendix

Lemma 3.3

Proof: Let *z* ∈ ℤ to be the ceiling of $\left|\frac{2\pi }{\psi_{i,j}^{+}-\psi_{i,j}^{-}}\right|$. The orbit of agent *i* can therefore be divided into *z* consecutive parts, with one of them to be totally covered by the rendezvous zone for *i*.

Without loss of generality, take the time point when agent *j* enters the rendezvous zone as *t* = 0, and the phase of *i* at this time as θ_*i*_(0). Before *i* and *j* rendezvous for the first time, *j* has a constant angular velocity ${\overset{∙}{\theta }}_{j}$, and enters rendezvous zone at the time points $\frac{2k\pi }{{\overset{∙}{\theta }}_{j}}$, for *k* ∈ ℕ_+_. The corresponding phases of *i* are $\theta_{i}\left(0\right)+2k\pi \frac{{\overset{∙}{\theta }}_{i}}{{\overset{∙}{\theta }}_{j}}=\theta_{i}\left(0\right)+kq\cdot \left(2\pi \right)$. If ∃*k* such that $\left(kq-\left\lfloor kq\right\rfloor \right)\cdot 2\pi +\theta_{i}\left(0\right)\in \left[\psi_{i,j}^{-},\psi_{i,j}^{+}\right]$, then *i* and *j* rendezvous.

An irrational *q* can be approximated arbitrarily close from above and below by a rational, such that there exists a rational number $\frac{a}{b}$ with *a, b* ∈ ℤ^+^ such that $\frac{a}{b}<q$, and $\frac{a+\frac{1}{z}}{b}>q$. If *k* = *b*, then $\left(kq-\left\lfloor kq\right\rfloor \right)\cdot 2\pi \in \left(0,\frac{2\pi }{z}\right)$. If *k* is an integer multiple of *b*, then the corresponding phase of *i* can match every of the *z* parts on the orbit. Since one of them is a subset of the rendezvous zone, agent *i* will eventually be in the rendezvous zone when *j* just enters the zone.

Theorem 4.1

Proof: Let $\vartheta_{i}\left(t\right)={\overset{∙}{\theta }}_{i}\left(t\right)-{\bar{\omega }}_{N}$, and $\Theta \left(t\right)=\overset{N}{\underset{i=1}{\sum }}|\vartheta_{i}\left(t\right)|$. The theorem's statement is equivalent to having $\underset{t\to \infty }{\lim}\Delta \left(t\right)=0$.

Pick randomly any pair (*i, j*) of agents in rendezvous, and consider the time interval between the time of their encounter, *t*^*rend*^, and the minimum time $\bar{t}$ between any of them departing the rendezvous region and any other pair of agents coming into rendezvous. For $t\in \left(t^{rend},\bar{t}\right)$,

$$|\vartheta_{i}\left(t\right)|+|\vartheta_{j}\left(t\right)|=|{\overset{∙}{\theta }}_{i}\left(t^{rend}\right)+{\overset{∙}{\theta }}_{j}\left(t^{rend}\right)-2{\bar{\omega }}_{N}|.$$

If ϑ_*i*_(*t*) and ϑ_*j*_(*t*) share the same sign, then

(23) $$\begin{array}{l} |\vartheta_{i}\left(t\right)|+|\vartheta_{j}\left(t\right)|=|\vartheta_{i}\left(t^{rend}\right)|+|\vartheta_{j}\left(t^{rend}\right)|. \end{array}$$

Thus note that Θ(*t*) remains constant for $t\in \left(t^{rend},\bar{t}\right)$ and is non-increasing over consecutive rendezvous events. It can be shown that Θ(*t*) cannot remain constant for ever.

While the network of oscillators has not yet reached consensus on their frequencies, there is bound to be at least one agent with angular velocity greater than ${\bar{\omega }}_{N}$, and at least one with angular velocity smaller than ${\bar{\omega }}_{N}$. If one pair of those oscillators with frequencies on opposite sides of ${\bar{\omega }}_{N}$ are in fact neighbors, then when they meet (and the falsification of the condition of Theorem 0.0.4 guarantees they will), Θ(*t*) will decrease. Indeed, while Θ(*t*)≠0, rendezvous between neighbors on opposite sides of ${\bar{\omega }}_{N}$ is bound to occur because the network is assumed to be connected. Agents on opposite sides of ${\bar{\omega }}_{N}$ still interact via shared neighbors: eventually pairs of adjacent agents with frequencies at different sides of ${\bar{\omega }}_{N}$ will appear. Otherwise, at least two isolated sub-graphs would exist in the network, which would contradict the connectivity assumption.

Theorem 5.2

Proof: Rearranging (16) in section 5 yields

(24) $$\begin{array}{l} m_{i}{\tilde{T}}_{i}-m_{j}{\tilde{T}}_{j}-\frac{\delta t_{i,j}}{T_{i}}{\tilde{T}}_{i}<\Delta {\tilde{t}}_{j,i}<m_{i}{\tilde{T}}_{i}-m_{j}{\tilde{T}}_{j}+\frac{\delta t_{j,i}}{T_{j}}{\tilde{T}}_{j}. \end{array}$$

For a valid $\Delta {\tilde{t}}_{j,i}$ to exist, we have that (i) the left hand side of (24) must be strictly less than the right hand side, and (ii) the range of (24) must overlap with $\left(-\frac{\delta t_{i,j}}{T_{i}}{\tilde{T}}_{i},\frac{\delta t_{j,i}}{T_{j}}{\tilde{T}}_{j}\right)$. The first part of the condition holds directly for both $\frac{\delta t_{i,j}}{T_{i}}$ and $\frac{\delta t_{j,i}}{T_{j}}$ are positive. The second part holds iff. the following inequalities hold for all possible α_*i,j*_ in the range of (15) in section 5.

(25) $$\begin{array}{l} \;m_{i}{\tilde{T}}_{i}-m_{j}{\tilde{T}}_{j}-\frac{\delta t_{i,j}}{T_{i}}{\tilde{T}}_{i}<\frac{\delta t_{j,i}}{T_{j}}{\tilde{T}}_{j},\\ \text{and }-\frac{\delta t_{i,j}}{T_{i}}{\tilde{T}}_{i}<m_{i}{\tilde{T}}_{i}-m_{j}{\tilde{T}}_{j}+\frac{\delta t_{j,i}}{T_{j}}{\tilde{T}}_{j}. \end{array}$$

Rearranging (25) provides us with

(26) $$\begin{array}{l} \frac{m_{j}-\frac{\delta t_{j,i}}{T_{j}}}{m_{i}+\frac{\delta t_{i,j}}{T_{i}}}<\frac{{\tilde{T}}_{i}}{{\tilde{T}}_{j}}<\frac{m_{j}+\frac{\delta t_{j,i}}{T_{j}}}{m_{i}-\frac{\delta t_{i,j}}{T_{i}}}. \end{array}$$

Since (26) holds for all α_*i,j*_ exists in (15) in section 5, it is clear that we should have

(27) $$\begin{array}{l} \frac{m_{j}-\frac{\delta t_{j,i}}{T_{j}}}{m_{i}+\frac{\delta t_{i,j}}{T_{i}}}<\frac{\omega_{j}+u_{j}-\eta_{s}}{\omega_{i}+u_{i}+\eta_{s}};\text{ and }\frac{\omega_{j}+u_{j}+\eta_{s}}{\omega_{i}+u_{i}-\eta_{s}}<\frac{m_{j}+\frac{\delta t_{j,i}}{T_{j}}}{m_{i}-\frac{\delta t_{i,j}}{T_{i}}}. \end{array}$$

As $\frac{m_{j}}{m_{i}}=\frac{\omega_{j}+u_{j}}{\omega_{i}+u_{i}}$, (27) is equivalent to

(28) $$\begin{array}{l} \eta_{s}<\frac{\omega_{i}+u_{i}}{m_{i}}\left(\frac{m_{i}\frac{\delta t_{j,i}}{T_{j}}+m_{j}\frac{\delta t_{i,j}}{T_{i}}}{m_{i}+m_{j}+|\frac{\delta t_{i,j}}{T_{i}}-\frac{\delta t_{j,i}}{T_{j}}|}\right), \end{array}$$

and $\eta_{s}<\frac{\delta t_{i,j}\left(\omega_{i}+u_{i}\right)}{m_{i}T_{i}}$ holds for $\frac{\delta t_{i,j}}{T_{i}}=\frac{\delta t_{j,i}}{T_{j}}$.

Theorem 5.5

Proof: For any

(29) $$\begin{array}{l} m_{i}\geq M_{i}=\lceil \frac{1-\lambda_{i}-\lambda_{j}+\lambda_{i}\lambda_{j}}{\lambda_{j}}\rceil , \end{array}$$

there is

(30) $$\begin{array}{r} \frac{1}{m_{i}+1-\lambda_{i}}>\frac{1-\lambda_{j}}{m_{i}}\text{ and }\frac{1}{m_{i}-\lambda_{i}}>\frac{1-\lambda_{j}}{m_{i}+1}. \end{array}$$

therefore the valid ranges of α_*i,j*_ to realize *m*_*i*_-1 scheme and (*m*_*i*_ + 1)-1 scheme overlap for *m*_*i*_ ≥ *M*_*i*_. Any α_*i,j*_ satisfying (21) in section 5 falls into some rendezvous scheme as long as both agents can finish at least one round within $\bar{T}$ amount of time.

## References

[B1] Athans M. Falb P. L. (2007). Optimal Control: An Introduction to the Theory and Its Applications. New York, NY: Dover Publications.

[B2] Bhadauria D. Tekdas O. Isler V. (2011). Robotic data mules for collecting data over sparse sensor fields. J. Field Robot. 28, 388–404. 10.1002/rob.20384

[B3] Buck J. Buck E. (1968). Mechanism of rhythmic synchronous flashing of fireflies. Science 159, 1319–1327. 10.1126/science.159.3821.1319 5644256

[B4] Dadush D. N. (2012). Integer programming, lattice algorithms, and deterministic volume estimation (PhD thesis), Georgia Institute of Technology, Atlanta, GA, United States.

[B5] Forgoston E. Billings L. Yecko P. Schwartz I. B. (2011). Set-based corral control in stochastic dynamical systems: making almost invariant sets more invariant. Chaos 21:013116. 10.1063/1.3539836 21456830 PMC4109835

[B6] Gazi V. Passino K. M. (2003). Stability analysis of swarms. IEEE Trans. Autom. Control 48, 692–696. 10.1109/TAC.2003.809765

[B7] Haller G. (2011). A variational theory of hyperbolic Lagrangian coherent structures. Phys. D 240, 574–598. 10.1016/j.physd.2010.11.010

[B8] Hsieh M. A. Hajieghrary H. Kularatne D. Heckman C. R. Forgoston E. Schwartz I. B. (2015). Small and adrift with self-control: using the environment to improve autonomy, in Robotics Research, eds Bicchi A. Burgard W. (Cham: Springer), 387–402.

[B9] Hsieh M. A. Mallory K. Forgoston E. Schwartz I. B. (2014). Distributed allocation of mobile sensing agents in geophysical flows, in Proceedings of American Control Conference (Portland, OR), 165–171.

[B10] Inanc T. Shadden S. Marsden J. (2005). Optimal trajectory generation in ocean flows, in Proceedings of American Control Conference (Portland, OR), 674–679.

[B11] Kularatne D. Bhattacharya S. Hsieh M. A. (2018). Going with the flow: a graph based approach to optimal path planning in general flows. Auton. Robots 42, 1369–1387. 10.1007/s10514-018-9741-6

[B12] Liu Y. Weisberg R. H. Vignudelli S. Mitchum G. T. (2016). Patterns of the loop current system and regions of sea surface height variability in the eastern gulf of mexico revealed by the self-organizing maps. J. Geophys. Res. Oceans 121, 2347–2366. 10.1002/2015JC011493

[B13] Papachristodoulou A. Jadbabaie A. (2006). Synchronization in oscillator networks with heterogeneous delays, switching topologies and nonlinear dynamics, in Proceedings of the 45th IEEE Conference on Decision and Control (San Diego, CA), 4307–4312.

[B14] Pikovsky A. Rosenblum M. Kurths J. (2001). Synchronization: A Universal Concept in Nonlinear Science. New York, NY: Cambridge University Press.

[B15] Sepulchre R. Paley D. Leonard N. (2004). Collective motion and oscillator synchronization, in Cooperative Control, eds Morse S. Leonard N. Kumar V. (Berlin; Heidelberg: Springer), 189–205.

[B16] Shadden S. C. Lekien F. Marsden J. E. (2005). Definition and properties of Lagrangian coherent structures from finite-time Lyapunov exponents in two-dimensional aperiodic flows. Phys. D 212, 271–304. 10.1016/j.physd.2005.10.007

[B17] Shuai J.-W. Durand D. M. (1999). Phase synchronization in two coupled chaotic neurons. Phys. Lett. A 264, 289–297. 10.1016/S0375-9601(99)00816-6

[B18] Sugihara R. Gupta R. K. (2011). Path planning of data mules in sensor networks. ACM Trans. Sensor Netw. 8, 1:1–1:27. 10.1145/1993042.1993043

[B19] Veronis G. (1966). Wind-driven ocean circulation, Part I and Part II. Deep Sea Res. 13:31 10.1016/0011-7471(66)90004-0

[B20] Wei C. Li C. Tanner H. G. (2018a). Synchronous rendezvous for periodically orbiting vehicles with very-low-range interactions, in Proceedings of American Control Conference (Milwaukee, WI), 1641–1646.

[B21] Wei C. Tanner H. G. Yu X. Hsieh M. A. (2019). Low-range interaction periodic rendezvous along Lagrangian coherent structures, in The American Control Conference (Philadelphia, PA).

[B22] Wei C. Yu X. Tanner H. G. Hsieh M. A. (2018b). Synchronous rendezvous for networks of active drifters in gyre flows, in The 14th International Symposium of Distributed Autonomous Robotic Systems (Boulder, CO).

[B23] Yuh J. Marani G. Blidberg D. R. (2011). Applications of marine robotic vehicles. Intell. Serv. Robot. 4:221 10.1007/s11370-011-0096-5

[B24] Zavlanos M. M. (2010). Synchronous rendezvous of very-low-range wireless agents, in Proceedings of the 49th IEEE Conference on Decision and Control (Atlanta, GA), 4740–4745.

[B25] Zeng X. Li Y. He R. Yin Y. (2015). Clustering of loop current patterns based on the satellite observed sea surface height and self-organizing map. Rem. Sens. Lett. 6, 11–19. 10.1080/2150704X.2014.998347

[B26] Zhang F. Marani G. Smith R. N. Choi H. T. (2015). Future trends in marine robotics [tc spotlight]. IEEE Robot. Autom. Mag. 22, 14–122. 10.1109/MRA.2014.2385561

